# Identification of epigenetic monotherapy candidates in taxane-resistant CRPC

**DOI:** 10.55730/1300-0152.2778

**Published:** 2025-11-05

**Authors:** Buse CEVATEMRE, İpek BULUT, Ezgi KARYEMEZ, Ceyda AÇILAN

**Affiliations:** 1 School of Medicine, Koç University, İstanbul, Turkiye; 2 Research Center for Translational Medicine, Koç University, İstanbul, Turkiye; 3 Graduate School of Health Sciences, Koç University, İstanbul, Turkiye

**Keywords:** Drug resistance, drug screen, epigenetic inhibitors, taxane, LSD1

## Abstract

**Background/aim:**

Taxane resistance remains a significant challenge in the effective treatment of castration-resistant prostate cancer (CRPC). Given the association of epigenetic regulation with chemotherapy resistance and cancer progression, this study aims to identify epigenetic vulnerabilities in two CRPC cell lines (DU145 and 22Rv1) established as resistant to two different taxanes, docetaxel (Dtx) and cabazitaxel (Cbz), using a small-molecule screening approach.

**Materials and methods:**

A small-molecule library targeting epigenetic regulators, including histone deacetylases (HDAC), histone methyltransferases, histone demethylases, bromodomain proteins, deoxyribonucleic acid methyltransferases, protein arginine deiminase, and histone acetyltransferase was utilized. Drug screening was performed on parental and taxane-resistant CRPC cell lines. Cell viability was assessed using the sulforhodamine B assay to identify compounds impairing the growth of resistant cells. Selected hits were further evaluated for their impact on colony-forming capacity using clonogenic assays, and cell death was confirmed by Annexin V/PI flow cytometry. Western blotting was used to assess histone modification marks (e.g., H3K27Ac, H3K4me2) and protein targets, including HDAC7 and lysine-specific demethylase 1 (LSD1). In combination studies, resistant cell lines were exposed to fixed-dose taxanes in combination with selected compounds. Combenefit software was used to generate synergy maps.

**Results:**

Screening results revealed that taxane-resistant CRPC cells remained susceptible to multiple epigenetic inhibitors rather than a single dominant class. Among the identified compounds, 4-Iodo-SAHA (HDAC inhibitor) and SP2509 (LSD1 inhibitor) emerged as cytotoxic agents, inducing cell death at levels comparable to those of parental cells. Further validation confirmed their efficacy in impairing cell viability and long-term survival in taxane-resistant CRPC cells, as demonstrated by Annexin V/PI flow cytometry. Both compounds induced epigenetic modulations consistent with their targets, reflected by increased histone marks (H3K27Ac for 4-Iodo-SAHA; H3K4me2 for SP2509), and were also associated with depletion of HDAC7 and LSD1, respectively. Combination assays demonstrated that both compounds potentiated Dtx activity and helped overcome resistance in taxane-resistant CRPC models.

**Conclusion:**

This study highlights epigenetic vulnerabilities in taxane-resistant CRPC and identifies 4-Iodo-SAHA and SP2509 as promising monotherapy candidates, demonstrating their ability to potentiate Dtx activity and overcome resistance.

## 1. Introduction

Castration-resistant prostate cancer (CRPC) remains a major clinical challenge as it represents the advanced stage of PC that no longer responds to androgen deprivation therapy (Le et al., 2023). In clinical practice, docetaxel (Dtx) is generally the first-line chemotherapy, while cabazitaxel (Cbz) is considered for patients who develop resistance to Dtx (Le et al., 2023). However, the development of resistance to these therapies significantly limits long-term survival in patients (de Porras et al., 2021). Understanding the mechanisms underlying taxane resistance and identifying novel therapeutic strategies are critical for improving treatment options in CRPC (Bumbaca and Li, 2018).

Epigenetic regulation, which encompasses DNA methylation, histone modifications, and chromatin remodeling, plays a pivotal role in cancer progression and drug resistance (Sharma et al., 2010; Wang et al., 2023). Aberrant epigenetic changes can activate prosurvival pathways and suppress apoptotic signals, enabling cancer cells to evade the cytotoxic effects of chemotherapy (Wang et al., 2023). Recent studies have highlighted the potential of targeting epigenetic regulators to reverse resistance and sensitize cancer cells to treatment. For instance, elevated histone deacetylase (HDAC) activity and hyperacetylation contributed to chemotherapy resistance in PC (Xu et al., 2018). More importantly, Dtx-resistant (DtxR) PC cells with higher levels of acyl-CoA and acetylated proteins were more sensitive to HDAC inhibitors compared with their Dtx-sensitive counterparts, suggesting that TSA and SAHA could be effective in treating chemotherapy-resistant mCRPC. However, vorinostat (SAHA)-based regimens were poorly tolerated due to excessive toxicities, leading to early study termination and highlighting the need for effective alternatives (Bradley et al., 2009; Schneider et al., 2012). To address this, we investigated the epigenetic vulnerabilities of two distinct CRPC cell lines (DU145 and 22Rv1), each resistant to a different taxane, Dtx and Cbz, using a small-molecule inhibitor library targeting key epigenetic regulators, including HDACs, histone acetyltransferases (HATs), histone methyltransferases (HMTs), DNA methyltransferases (DNMTs), and bromodomain proteins (BRDs). Through this approach, 4-Iodo-SAHA (HDAC inhibitor) and SP2509 [Lysine-Specific Demethylase 1 (LSD1) inhibitor] emerged as promising monotherapy candidates, demonstrating potent cytotoxic effects in taxane-resistant CRPC cells, with effectiveness comparable to that of parental cells, and further potentiating Dtx response in resistant sublines in combination settings.

## 2. Materials and methods

### 2.1. Establishment of taxane-resistant cell lines

Docetaxel (Dtx) and Cabazitaxel (Cbz) resistant (R) CRPC cell lines were generated from parental (P) DU145 and 22Rv1 cells through 72h exposures to increasing concentrations of Dtx (01885, Merck) and Cbz (SML2487, Merck). Between each dose increment, cells were allowed to recover for 2–3 weeks to ensure survival and adaptation, with the entire process taking 8–12 months. The resistance of these cell lines was confirmed by performing dose–response viability assays, including SRB and clonogenic assays (described below), which demonstrated higher colony numbers. Herein, the designations DtxR and CbzR may be combined with cell line designations, e.g., DU145-DtxR or 22Rv1-CbzR.

### 2.2. Epigenetic drug screening

The epigenetic drug library (11076) was purchased from Cayman Chemical. Compounds were provided as 10 mM stock solutions dissolved in dimethyl sulfoxide (DMSO). Intermediate stocks were prepared at a concentration of 125 μM. On the day of treatment, cells were exposed to the drugs using these intermediate stocks. Cells were seeded in 96-well plates the day before treatment, with DU145 cells at 4000 cells/well and 22Rv1 cells at 7500 cells/well. Twenty-four h after seeding, cells were exposed to a 5-μM dose of each inhibitor from the epigenetic drug library for 72 h. At the end of the treatment period, cell viability was assessed using the sulforhodamine B (SRB) assay. This 5-μM dose was selected based on its widespread use and suggested applicability in phenotypic screening protocols using epigenetic compound libraries (Wei et al., 2025), particularly as implemented with the Cayman Chemical library in Schniewind et al. (2024). This choice was also supported by our prior experience using the same concentration in a related study (Cevatemre et al., 2024). The DU145-DtxR cell line was previously used in our earlier study to evaluate the effects of an epigenetic inhibitor–taxane combination (Cevatemre et al., 2024). In this study, only newly generated monotherapy data (epigenetic inhibitors alone) for this cell line are presented, and all figures were generated anew.

### 2.3. SRB assay

DU145 and 22Rv1 cells were seeded at densities of 4000 and 7500 cells/well, respectively, in 96-well plates. Following the 72-h treatment, cells in the 96-well plates were fixed by gently adding trichloroacetic acid (TCA, Sigma-Aldrich T6399) to each well at a final concentration of 10% (w/v), then incubating at 4 °C for 1 h. Plates were then washed with deionized water to remove excess TCA and air-dried. Cells were stained with 0.4% (w/v) SRB (Santa Cruz, sc-253615) dye in 1% acetic acid for 30 min at room temperature. Unbound dye was removed by washing with 1% acetic acid, and the plates were air-dried again. Bound SRB dye was solubilized in 10 mM Tris base, and absorbance was measured at 540 nm using a microplate reader. Absorbance (Abs) values were normalized to untreated controls. The percentage of cell viability was calculated using the formula % Cell Viability = [(Abs of treated cells − blank)/(Abs of untreated cells − blank)] × 100.

For dose–response validation studies, the maximum concentration was set at 10 μM to maintain DMSO levels at or below 0.1%, given that the compounds were supplied as 10 mM stock solutions. This ensured that solvent-related cytotoxicity was minimized during treatment.

### 2.4. CellTiter-Glo (CTG) luminescent cell viability assay

DU145 and 22Rv1 cells were seeded at densities of 4000 and 7500 cells/well, respectively, in 96-well plates. Following a 72-h treatment, cell viability was determined using the CTG Luminescent Cell Viability Assay (Promega, G7570) according to the manufacturer’s instructions. Briefly, CTG reagent was added directly to each well at a volume equal to one-tenth the volume of culture medium. Plates were incubated for 20 min at 37 °C to induce cell lysis and stabilize the luminescent signal. Luminescence was then measured using a plate reader. Relative luminescence units were normalized to the untreated control, and cell viability was calculated as described for the SRB assay. For synergy analysis, viability data were imported into Combenefit software (Di Veroli et al., 2016). Dose–response matrices of 4-Iodo-SAHA and Dtx were analyzed using the Highest Single Agent (HSA) reference model, and three-dimensional (3D) interaction surfaces were generated.

### 2.5. Clonogenic assay

The clonogenic assay was performed to evaluate resistance by assessing the long-term survival and proliferation ability of cells following taxane exposure. CRPC cells were seeded in 12-well plates at optimized densities (500 cells for DU145 and 1000 cells for 22Rv1) and allowed to adhere overnight. Cells were treated with Dtx and Cbz for 72 h. Following treatment, cells were washed with phosphate-buffered saline (PBS; Biowest, L0616) to remove residual drugs and cultured in fresh drug-free medium for 10–14 days to allow colony formation. Colonies were fixed with ice-cold methanol (ISOLAB, 947.046) at −20 °C for 20 min and stained with a 0.01% crystal violet (w/v) solution (Thermo, B21932.36) for 30 min. Results were normalized to untreated control wells, and the surviving fraction was calculated to confirm resistance.

### 2.6. Annexin V/PI flow cytometry using the Muse cell analyzer

Apoptosis was assessed using the Muse Annexin V & Dead Cell Kit (Luminex, MCH100105) according to the manufacturer’s instructions with minor modifications. DU145 and 22Rv1 parental (P), DtxR, and CbzR cells were seeded in 6-well plates and treated with 4-Iodo-SAHA (2.5 μM) for 48 h. Cells were harvested, washed once with PBS, and resuspended in PBS supplemented with 1% fetal bovine serum (FBS) to obtain a suspension containing ~250 cells/μL. For staining, 75 μL of Muse Annexin V/Dead Cell reagent was mixed with 75 μL of the cell suspension. Samples were incubated for 20 min at room temperature in the dark and then analyzed using the Muse Cell Analyzer.

### 2.7. Histone extraction and western blotting

DU145 and 22Rv1 parental (P), DtxR, and CbzR cells (1 × 10^6^) were seeded into 6-well plates and treated with 4-Iodo-SAHA (1 μM) for 6 h. At the end of incubation, cells were collected by trypsinization. For histone extraction, the pellet was resuspended in PBS containing 2 mM phenylmethylsulfonyl fluoride (PMSF) and 0.5% Triton X-100 and incubated on ice for 10 min. Samples were centrifuged at 6500 × g for 10 min at 4 °C. The pellet was resuspended in 0.2 N HCl, and the mixture was incubated overnight at 4 °C. After centrifugation (6500 × g, 10 min, 4 °C), the histone-containing supernatant was neutralized with 0.1 N NaOH. Protein concentration was measured with the Pierce BCA Protein Assay Kit. Proteins (0.5–1 μg) were separated on 4–15% Tris-Glycine SDS gels and transferred to polyvinylidene fluoride (PVDF) membranes using a semidry transfer system (25 V, 1 A, 30 min; Bio-Rad). Membranes were blocked with 5% bovine serum albumin (BSA) for 1 h at room temperature, washed with Tris-Buffered Saline with Tween 20, and incubated overnight at 4 °C with Ac-H3K27 (CST, 8173), H3K4me2 (CST, 9725), and histone H3 (CST, 9715) antibodies. After washing, membranes were incubated with horseradish peroxidase (HRP)-conjugated secondary antibody for 1 h at room temperature, and signals were detected with the LI-COR Odyssey imaging system.

For HDAC7 detection, cells were collected by trypsinization and lysed in RadioImmunoPrecipitation Assay (RIPA) buffer on ice for 30 min. Lysates were centrifuged at 16,000 × g for 15 min at 4 °C, and the protein-containing supernatant was collected. Protein concentration was determined using the Pierce BCA Protein Assay Kit. A total of 20–30 μg protein was loaded per lane of a 4–15% Tris-Glycine SDS gel, and electrophoresis was performed at 80 V for 1–1.5 h. Proteins were transferred to PVDF membranes (Bio-Rad, 1620177) using a Bio-Rad transfer system at 200 mA for 2 h at 4 °C. Subsequent blocking, washing, antibody incubation, and detection steps were performed as described for histone blots, using HDAC7 (Santa Cruz, sc-74563) antibody.

### 2.8. Statistical analysis

Statistical analyses were performed using GraphPad Prism 10. All experiments were conducted in at least duplicates, and data are presented as the mean ± standard error of the mean (SEM). Group comparisons were analyzed using two-way analysis of variance (ANOVA) to determine statistical significance. P-values less than 0.05 were considered statistically significant and are indicated in the figures as follows: p < 0.05 (*), p < 0.01(**), p < 0.001 (***), and p < 0.0001 (****). Graphs and statistical results were generated using GraphPad Prism 10.

## 3. Results

### 3.1. Validation of taxane resistance in CRPC cells

**Figure 1:**
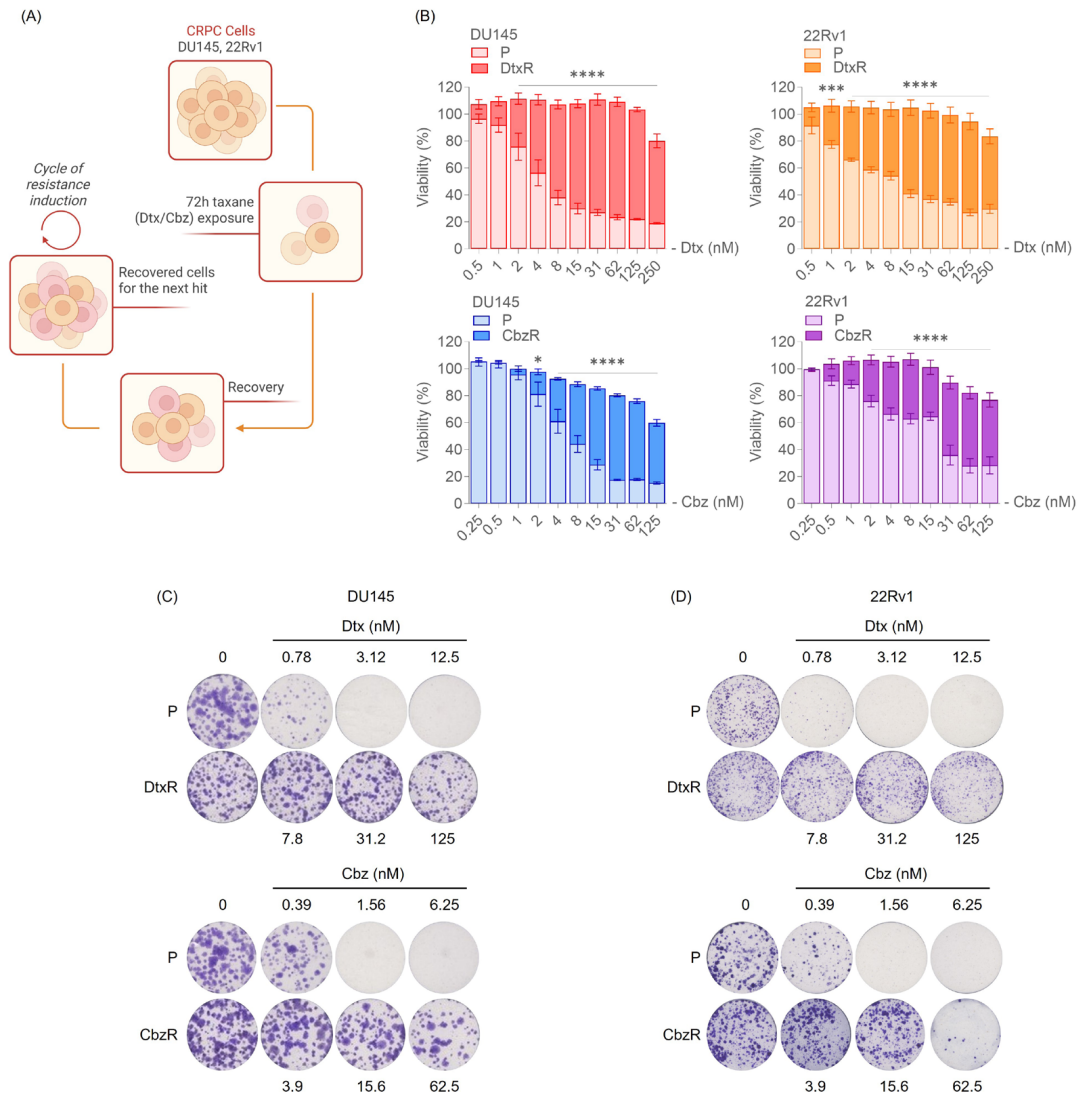
Establishment and characterization of taxane-resistant CRPC cells. (A) Schematic representation of the process used to establish taxane-resistant cell lines, created using BioRender. CRPC cells (DU145, 22Rv1) were subjected to repeated cycles of Dtx or Cbz treatment (72 h), followed by drug-free recovery, to generate resistant sublines. (B) Resistance was characterized by higher viability of taxane-resistant cells (DtxR and CbzR) than parental (P) cells at matched taxane concentrations, as assessed by SRB assays. Cells were treated with increasing concentrations of Dtx or Cbz for 72 h. Data represent mean ± SEM from two independent biological replicates, each with technical duplicates. Statistical analysis was performed using two-way ANOVA followed by Sidak’s multiple comparisons test. Significance levels: p < 0.05 (*), p < 0.01 (**), p < 0.001 (***), p < 0.0001 (****) for comparisons between parental and resistant cells at the same drug concentration. (C–D) Clonogenic assays further confirm the resistance phenotype in DU145 (C) and 22Rv1 (D) models. Resistant cells maintained colony formation capacity at doses that eliminated colonies in parental cells. Representative well images are shown.

The resistance of docetaxel- (DtxR) and cabazitaxel-resistant (CbzR) CRPC cell lines, compared with their parental counterparts (P), was confirmed using two different viability assays (Figure 1). In the SRB assay, resistant cells exhibited significantly higher viability compared with their parental counterparts when treated with increasing concentrations of Dtx or Cbz. Similarly, clonogenic assays showed that resistant cells formed more viable colonies after taxane treatment, indicating enhanced survival and proliferative capacity under drug exposure. These results demonstrate that the resistant cell lines are less sensitive to taxanes, making them suitable models for investigating epigenetic vulnerabilities.

### 3.2. Identification of epigenetic vulnerabilities in taxane-resistant cells

**Figure 2:**
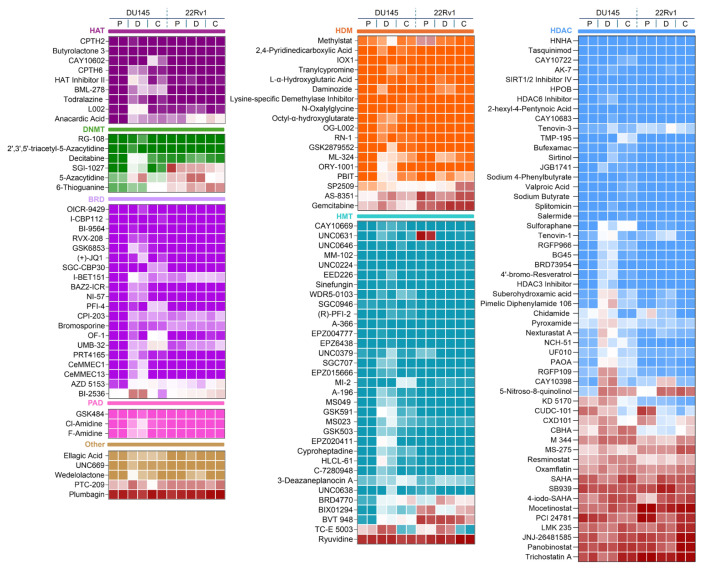
Epigenetic drug screening on taxane-sensitive and resistant CRPC cells. A color-coded heatmap showing the response of P, DtxR (D), and CbzR (C) CRPC cell lines (DU145 and 22Rv1) to a library of epigenetic inhibitors targeting specific regulators: HAT, HDM, DNMT, BRD, HMT, PAD, and HDAC. Each class is visually separated by a distinct background color behind its label (e.g., dark purple for HAT, orange for HDM). Cell viability was assessed using the SRB assay after 72 h of drug treatment at 5 μM and is shown as a percentage relative to the untreated control. The assigned group-specific colors indicate high cell viability, while low viability is represented by shades of red, indicating significant reductions in cell survival.

Taxane-resistant cells (DtxR and CbzR) exhibited distinct sensitivity profiles, with notably consistent reductions in viability observed for HDAC inhibitors (Figure 2). Specifically, several compounds from this class reduced cell viability by more than 50% in nearly one-third of the tested epigenetic drugs. On the other hand, when focusing on epigenetic drugs that showed increased sensitivity specifically in taxane-resistant cells, no single dominant class was identified. Instead, such phenotypes were observed across all epigenetic classes (Figure 2). To further validate the robustness and reliability of our findings, hierarchical clustering was employed to complement the heatmap visualization by uncovering patterns and relationships across the entire dataset that might not be immediately apparent. This approach systematically grouped compounds based on their overall effects across parental and taxane-resistant cell lines, allowing for a more nuanced comparison of sensitivity patterns.

**Figure 3:**
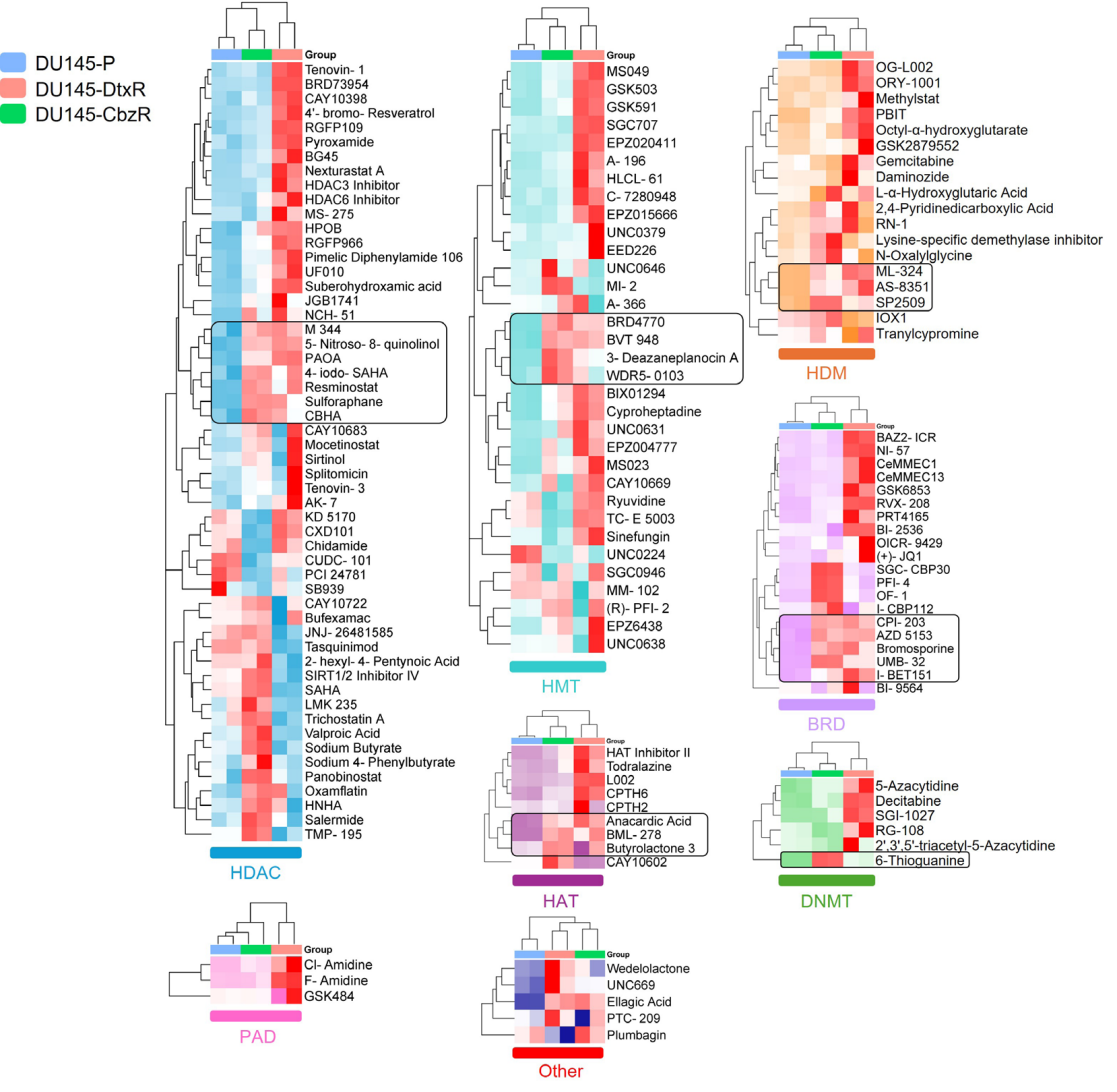
Hierarchical clustering of cell viability responses to epigenetic inhibitors in DU145 parental and taxane-resistant cell lines. Hierarchical clustering of cell viability data for P (blue), DtxR (pink), and CbzR (green) DU145 cells. Cell viability was assessed using the SRB assay after 72 h of treatment (5 μM), and results were normalized to untreated controls. Clustering was performed and visualized via SRplot. Dendrogram branches indicate the similarity of drug responses among the cell lines. Compounds enclosed in black boxes exhibit similar efficacy in both DtxR and CbzR cells. These compounds were shortlisted for cross-validation across both DU145 and 22Rv1 cell models to prioritize candidates independent of cell-line differences.

**Figure 4:**
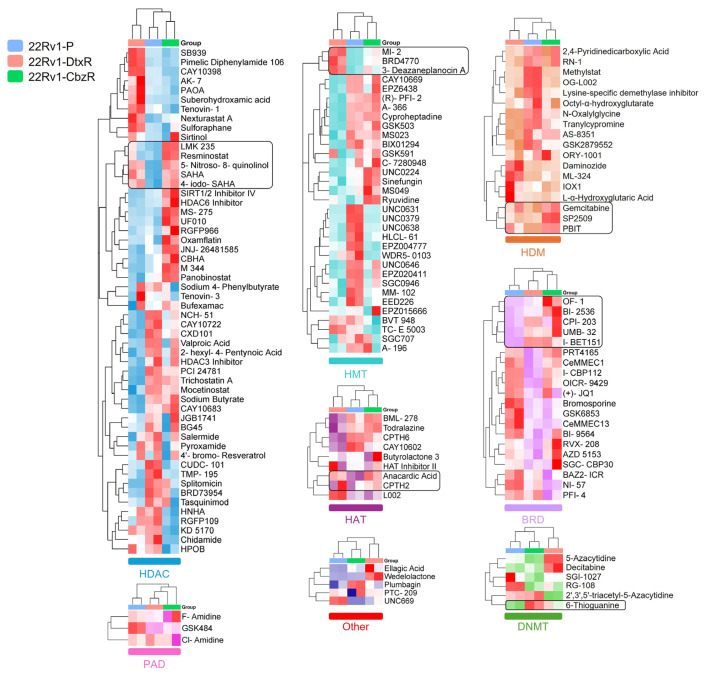
Hierarchical clustering of cell viability responses to epigenetic inhibitors in 22Rv1 parental and taxane-resistant cell lines. Hierarchical clustering of cell viability data for P (blue), DtxR (pink), and CbzR (green) 22Rv1 cells. Cell viability was assessed using the SRB assay after 72 h of treatment (5 μM), and results were normalized to untreated controls. Clustering was performed and visualized via SRplot. Dendrogram branches indicate the similarity of drug responses among the cell lines. Compounds enclosed in black boxes exhibit similar efficacy in both DtxR and CbzR cells. These compounds were shortlisted for cross-validation across both DU145 and 22Rv1 cell lines to prioritize candidates independent of cell-line differences.

**Figure 5:**
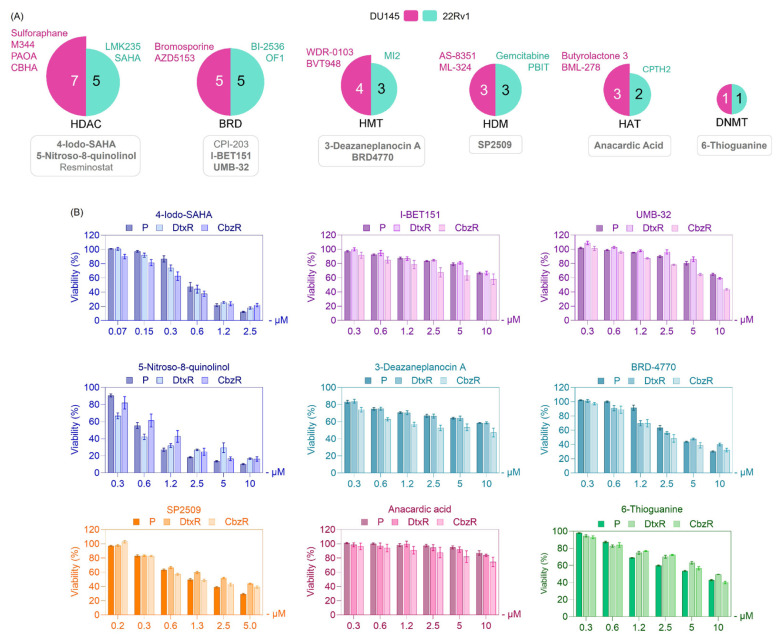
Validation of selected epigenetic inhibitors that reduce viability as monotherapy in CRPC cells. (A) Epigenetic compounds that reduced viability in taxane-resistant DU145 and 22Rv1 cells were selected from the initial screen and classified by their targets: HDAC, BRD, HMT, HDM, HAT, and DNMT. These compounds correspond to the cell-based hits indicated within black boxes in Figures 3 (DU145) and 4 (22Rv1). Pie charts illustrate growth-inhibitory compounds per target class, with chart sizes proportional to the number of epigenetic compounds. DU145 hits are shown in pink and 22Rv1 hits in turquoise. Shared hits between the two cell lines are listed in the boxes below each pie chart. Compounds shown in bold were selected for further validation based on their inhibitory effects across multiple doses. (B) Dose–response viability assays were performed for the inhibitors shown in bold in panel A, using DU145-P, DtxR, and CbzR cells. Cells were treated with increasing concentrations of each compound for 72 h, and viability was assessed using the SRB assay. Data represent mean ± SEM from at least two biological replicates, each performed in technical triplicate. Compounds 4-Iodo-SAHA and SP2509 were further selected for secondary validation using clonogenic survival assays, as shown in Figure 6. Their selection was based on efficacy in reducing cell viability and distinct epigenetic targets, as detailed in the main text.

We aimed to identify compounds that, as monotherapy, could eliminate taxane-resistant cells as effectively as parental cells. However, hierarchical clustering of DU145 and 22Rv1 cell lines treated with epigenetic inhibitors did not identify a single main group. Instead, effective compounds were distributed across multiple epigenetic target groups, including histone methyltransferase (HMT), histone demethylase (HDM), deoxyribonucleic acid methyltransferase (DNMT), and BRD inhibitors, with HDAC inhibitors emerging as the leading class, as demonstrated in Figures 3–5. These findings highlight the heterogeneity of epigenetic vulnerabilities in taxane-resistant CRPC cells.

### 3.3. Validation of identified epigenetic inhibitors in taxane-resistant CRPC cells

To confirm the findings from the library screen, selected epigenetic inhibitors (marked with rectangular boxes in Figures 3 and 4) that showed potential efficacy were further assessed. Validation experiments revealed that these compounds inhibited the growth of resistant cells to a similar extent as parental cells (Figure 5).

**Figure 6:**
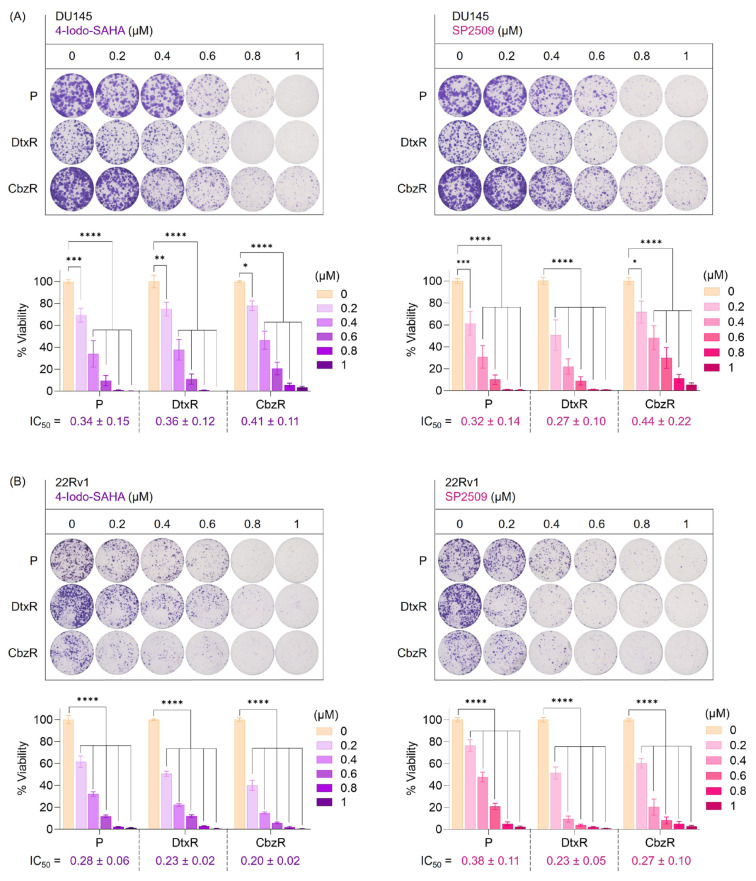
Taxane-resistant PC cells remain vulnerable to 4-Iodo-SAHA and SP2509 treatment. Long-term effects of 4-Iodo-SAHA (HDAC inhibitor) and SP2509 (LSD1 inhibitor) on cell survival were assessed by clonogenic assays in parental and taxane-resistant PC models, confirming their effects observed in short-term assays (Figure 5B; 72-h SRB assay). Representative images (top) and quantification of colony areas (bottom) are shown. Despite acquired taxane resistance, both compounds suppressed clonogenic survival in a dose-dependent manner across all cell types. (A) DU145-P, DtxR, and CbzR cells were treated with increasing concentrations (0.2–1 μM) of 4-Iodo-SAHA (left) or SP2509 (right) for 72 h. Calculated IC_50_ values (mean ± SD) are shown beneath each corresponding cell line in the bar graphs. (B) The same treatments were applied to 22Rv1-P, DtxR, and CbzR cells. Calculated IC_50_ values (mean ± SD) are shown beneath each corresponding cell line in the bar graphs. Colony areas were quantified using ImageJ. Data represent mean ± SEM from at least two independent biological replicates, each with technical duplicates. Statistical analysis was performed using two-way ANOVA followed by Dunnett’s multiple comparisons test. Significance levels: p < 0.05 (*), p < 0.01 (**), p < 0.001 (***), p < 0.0001 (****) compared with corresponding ‘0 μM’ controls.

To further validate the cytotoxic potential of selected hits, we prioritized compounds based on both their efficacy profiles and their representation within the screening data. Among the most promising candidates, we selected 4-Iodo-SAHA from the HDAC inhibitor class, which had the highest number of hits across both cell lines (Figure 5A). While 5-Nitroso-8-quinolinol also showed cytotoxicity, its response pattern differed notably in resistant cells, prompting the selection of 4-Iodo-SAHA for validation. As a mechanistically distinct candidate, we additionally focused on SP2509, a selective LSD1 inhibitor, for its potent growth-inhibitory effect at low concentrations compared with other compounds (Figure 5B). These two compounds were subsequently tested in clonogenic assays to assess their long-term effects on cell survival in both parental and taxane-resistant PC models (Figure 6). Clonogenic assays demonstrated that both 4-Iodo-SAHA and SP2509 markedly suppressed colony-forming capacity in DU145 and 22Rv1 cells, regardless of taxane resistance status (Figure 6A–B). Treatment with increasing concentrations of either compound resulted in a dose-dependent reduction in clonogenic survival across the parental, DtxR, and CbzR sublines. Complete inhibition of colony formation was achieved at 0.8–1 μM, underscoring the potent and consistent efficacy of these agents. Comparable reductions in viability between parental and resistant cells suggest that their cytotoxic effects are preserved despite acquired taxane resistance. These results highlight the potential of 4-Iodo-SAHA and SP2509 as effective monotherapies for treatment-refractory CRPC.

### 3.4. Cellular effects of 4-Iodo-SAHA in DtxR cells

**Figure 7:**
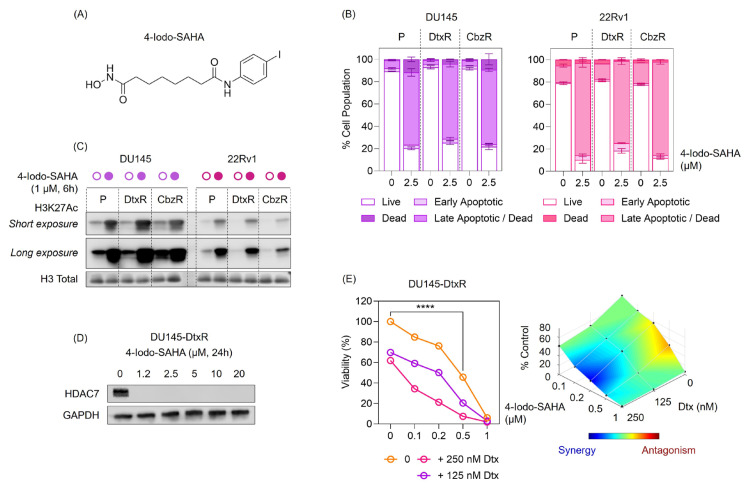
4-Iodo-SAHA induces apoptosis, elevates histone acetylation, suppresses HDAC7, and resensitizes DtxR cells. (A) Chemical structure of 4-Iodo-SAHA. (B) Annexin V/PI flow cytometry profiles showing redistribution from live to early/late apoptotic and dead fractions following 4-Iodo-SAHA treatment (2.5 μM, 48 h) in DU145 and 22Rv1 parental (P), DtxR, and CbzR sublines. Data represent mean ± SEM from two replicates. (C) Western blot detection of H3K27Ac following histone extraction from DU145 and 22Rv1 parental, DtxR, and CbzR sublines treated with 4-Iodo-SAHA (1 μM, 6 h). Both short and long exposures are presented; H3 Total was used as the loading control. Open circles indicate control; filled circles indicate 4-Iodo-SAHA treatment. (D) Western blot analysis of HDAC7 protein levels in DU145-DtxR cells after 4-Iodo-SAHA treatment (1.2–20 μM, 24 h). GAPDH was the loading control. Experiments were repeated twice; a representative blot is shown. (E) Left: Dose-response curves of DU145-DtxR cells treated with 4-Iodo-SAHA alone (0, 0.1–1 μM) or in combination with fixed doses of Dtx (125 or 250 nM). Statistical analysis was performed using two-way ANOVA followed by Dunnett’s multiple comparisons test. Significance level: p < 0.0001 (****) versus the corresponding “4-Iodo-SAHA only (0)” group. Right: Interaction surface generated with Combenefit (HSA model) for the “4-Iodo-SAHA × Dtx” combination. Heatmap colors indicate the interaction effect (blue, synergy; red, antagonism). Data represent mean ± SEM from two biological replicates, each performed with technical duplicates.

Building on the validation in Figure 6, we next profiled the cellular effects of 4-Iodo-SAHA and SP2509 (Figure 7A; Supplemental Figure A). Annexin-V/PI analysis showed a redistribution from viable to early/late apoptotic fractions in DU145 and 22Rv1 following 4-Iodo-SAHA treatment (Figure 7B), indicating that cytotoxicity is accompanied by apoptosis. Immunoblotting revealed increases in H3K27 acetylation in both models (Figure 7C), consistent with HDAC inhibition by 4-Iodo-SAHA. Prompted by previous work showing that hydroxamic-acid HDAC inhibitors selectively suppress HDAC7 (Dokmanovic et al., 2007), we examined HDAC7 protein levels and found that 4-Iodo-SAHA abolished HDAC7 protein at all concentrations tested (Figure 7D). Together with the increase in H3K27Ac (Figure 7C), these findings support an HDAC-axis mechanism of action. Moreover, combination assays demonstrated that 4-Iodo-SAHA overcame Dtx resistance in DU145-DtxR cells, as evidenced by enhanced growth inhibition and a synergy map generated with the Combenefit HSA model (Figure 7E).

In parallel, SP2509 induced cytotoxicity in taxane-resistant cells (Supplemental Figure B). This effect was accompanied by canonical evidence of LSD1 inhibition, including increased H3K4me2 levels (Supplemental Figure C) and reduced LSD1 protein levels (Supplemental Figure D). In combination assays, SP2509 further enhanced Dtx-mediated growth inhibition, with synergy maps generated using the Combenefit HSA model supporting synergistic effects (Supplemental Figure E). Collectively, these findings support both 4-Iodo-SAHA and SP2509 as potential monotherapy candidates in taxane-resistant PC, while also demonstrating that each compound potentiated Dtx activity and contributed to overcoming resistance.

## 4. Discussion

In this study, we investigated the epigenetic vulnerabilities of taxane-resistant CRPC cell lines using a small-molecule inhibitor library targeting key epigenetic regulators (Figures 1–4). Our findings demonstrated that taxane-resistant cells exhibit sensitivity profiles similar to those of their parental counterparts, with effective compounds distributed across multiple epigenetic inhibitor classes, including HDAC, HAT, BRD, and HDM inhibitors, rather than a single dominant class. Validation experiments supported our cluster analysis and screening results, confirming the sensitivity of resistant cells to the identified epigenetic drugs across various classes (Figure 5). These results highlight the potential of targeting epigenetic mechanisms to overcome resistance and improve therapeutic outcomes in CRPC. 4-Iodo-SAHA and SP2509 emerged as epigenetic drugs exhibiting cytotoxic effects beyond growth inhibition (Figure 6). Their cell-killing efficacy, comparable to that observed in parental cells, highlights their potential as promising candidates for drug-resistant advanced cancers. 4-Iodo-SAHA (1 μM) has been identified as an inhibitor of osteogenesis and adipogenesis (Dhaliwal et al., 2018). It also caused significant Golgi dispersal and was classified as a Golgi disruptor (Gendarme et al., 2017). Additionally, treatment of diffuse intrinsic pontine glioma (DIPG) neurospheres with BGB324 (an AXL inhibitor) in combination with varying doses of 4-Iodo-SAHA (200–500 nM) synergistically reduced DIPG cell viability (Meel et al., 2020). While the efficacy of SAHA (Vorinostat) has been extensively studied and approved by the United States Food and Drug Administration for the treatment of cutaneous T-cell lymphoma (Bubna, 2015), the potential of 4-Iodo-SAHA has not been established, not only in resistant cells but even in PC. Therefore, this study is the first to demonstrate its potential as a monotherapy in taxane-resistant PC.

SP2509 has been relatively well-studied, particularly in a study that found LSD1 to be upregulated in PC and associated with prognosis, promoting cancer cell survival by downregulating FBXW7 protein levels (Qin et al., 2021). Treatment with SP2509 (1 μM) suppressed cell survival by blocking the LSD1–FBXW7 interaction. It caused LSD1 depletion, which led to the upregulation of FBXW7 expression levels. Furthermore, SP2509 recapitulated the effects of LSD1 ribonucleic acid interference (RNAi) on cell viability. SP2509 (1 μM) also reduced cell viability and consistently induced apoptosis across a panel of PC cell lines (Wang et al., 2020), supporting our findings and further highlighting its potential as a therapeutic agent in PC. TBX2-driven repression of androgen receptor and activation of glucocorticoid receptor led to enzalutamide resistance. At the same time, SP2509 disrupted both TBX2-LSD1 and TBX2-GR protein–protein interactions, highlighting a unique mode of action for this compound in CRPC (Dutta et al., 2024). SP2509 efficacy extends to other tumor types as well. For instance, LSD1 was overexpressed in retinoblastoma cells, promoting their survival, while SP2509 similarly inhibited growth in vitro (0.06–5 μM) and in vivo, partly via β-catenin pathway suppression (Jiang et al., 2022). Additionally, using a spindle and kinetochore-associated complex lung cancer cell line [A549 with a signal transducer and activator of transcription 3 (STAT3)-driven luciferase reporter], SP2509 was identified as a STAT3 signaling inhibitor (2.5–10 μM) that suppressed cell growth (IC_50_ = 3.085 μM for A549) and induced apoptosis through Janus kinase (JAK)/STAT3 modulation (Zhen et al., 2021). In clear cell renal cell carcinoma (ccRCC), SP2509 significantly suppressed cell proliferation by inducing G1/S cell cycle arrest and promoting H3K4me2 accumulation (at concentrations ranging from 0.125 to 8 μmol/L; –log_2_ scale), while also inhibiting (15 mg/kg) clear cell renal cell carcinoma (ccRCC) xenograft tumor growth in vivo (Zhu et al., 2019). SP2509 (at concentrations ranging from 0.03 to 1 μM) enhanced the sensitivity of established and patient-derived ovarian cancer cells to chemotherapy, reducing cell viability and clonogenic survival while inducing apoptosis (Chen et al., 2024). However, its efficacy in a taxane-resistant setting has not been demonstrated. Our study provides evidence that SP2509 is cytotoxic against taxane-resistant PC cells, killing them at levels comparable to those of parental cells.

In a study, the combination of azacitidine followed by Dtx, along with growth factor support and fixed prednisone, demonstrated activity in mCRPC patients whose disease progressed during or after Dtx therapy (Singal et al., 2015). The combination of vorinostat and Dtx was poorly tolerated, with excessive dose-limiting toxicities leading to early study termination in patients, including those with CRPC, highlighting the need to identify viable monotherapy options (Schneider et al., 2012). A phase II trial was designed to evaluate the efficacy of vorinostat in chemotherapy-pretreated patients with mCRPC (Bradley et al., 2009). However, vorinostat was associated with significant toxicities, limiting efficacy assessment in this patient population and further emphasizing the need for newer agents with a more favorable toxicity profile and substantial supportive preclinical data.

While our results support the rationale for epigenetic monotherapies in CRPC, many epigenetic drugs exhibit paninhibitory activity, which can indiscriminately affect multiple members of an enzyme family, increasing the likelihood of unintended interactions and adverse effects (Shah, 2019; Dai et al., 2024). In this context, although 4-Iodo-SAHA and SP2509 demonstrated promising cytotoxicity in both parental and taxane-resistant cells, their off-target profiles warrant further consideration. For SP2509, previous work demonstrated that the compound induces cell death in LSD1-knockout cells, indicating that its cytotoxic activity is independent of LSD1 inhibition (Sonnemann et al., 2018). It was also noted that the core structure of SP2509 corresponds to a pan-assay interference compounds (PAINS) motif (Mould et al., 2015), which is capable of interacting non-specifically with diverse proteins beyond the intended target. In addition, an LSD1-independent off-target effect involving a potential mitochondrial component has been suggested, further underscoring the need to clarify the precise mechanism of action of SP2509 (Tokarsky et al., 2022). Our analyses showed that SP2509 alone exhibited marked cytotoxicity in DtxR cells (Supplemental Figure B). This effect was accompanied by a dose-dependent increase in H3K4me2 levels (Supplemental Figure C) and a reduction in LSD1 protein levels (Supplemental Figure D), consistent with canonical inhibition and target engagement. Moreover, in combination with Dtx, SP2509 produced a synergistic effect in resistant cells (Supplemental Figure E), indicating that LSD1 targeting enhances taxane sensitivity and overcomes resistance. Thus, while SP2509 behaves as an effective probe of the LSD1 axis in our system, its known off-target liabilities and LSD1-independent cytotoxicity argue that validation with more selective LSD1 inhibitors and orthogonal genetic approaches will be essential.

Our study provides new insights into the epigenetic vulnerabilities of taxane-resistant CRPC. It highlights the potential of targeting epigenetic regulators, with 4-Iodo-SAHA and SP2509 emerging as promising monotherapy candidates warranting further investigation in advanced disease settings. Although the current study focused on two-dimensional (2D) in vitro models, future evaluation of these compounds in 3D spheroids or in vivo xenograft systems could further strengthen the translational relevance of our findings. It is also worth noting that, given existing concerns about potential off-target effects, particularly with SP2509, comprehensive mechanistic studies will be essential to assess the specificity and safety of these agents prior to clinical translation in drug-naive or drug-resistant CRPC.

## Supplementary material

Supplemental Figure Cellular effects of SP2509.
(A) Chemical structure of SP2509. (B) Annexin V/PI flow cytometry profiles showing redistribution from live to early/late apoptotic and dead fractions following SP2509 treatment (6 μM, 72 h) in 22Rv1 parental (P), DtxR, and CbzR cells. Data represent mean ± SEM from two replicates. (C) Western blot detection of H3K4Me2 following histone extraction from 22Rv1-DtxR cells treated with increasing concentrations of SP2509 (1.5–25 μM, 6 h). H3 Total was used as the loading control. (D) Left: Western blot detection of LSD1 protein in DU145-DtxR cells treated with SP2509 (1.5–25 μM, 24 h). GAPDH was the loading control. Rigt: Quantification of LSD1 protein levels normalized to GAPDH, shown relative to untreated control (0). Statistical analysis was performed using one-way ANOVA followed by Dunnett’s multiple comparisons test. Significance levels: p < 0.05 (*), p < 0.01 (**) compared with corresponding ‘0 μM’ controls. (E) Upper panel: Dose-response curves of 22Rv1-DtxR and DU145-DtxR cells treated with SP2509 alone (0, 0.3–12.5 μM) or in combination with fixed doses of Dtx (125–500 nM). Lower panel: Interaction surface generated with Combenefit (HSA model) for the “SP2509 × Dtx” combination. Heatmap colors indicate the interaction effect (blue, synergy; red, antagonism).
