# Supplementary figures and images for: Identification of epigenetic monotherapy candidates in taxane-resistant CRPC
Source: Turk J Biol. 2025 Nov 5;49(7):757–69. doi: 10.55730/1300-0152.2778 (PMC12768438; doi:10.55730/1300-0152.2778)

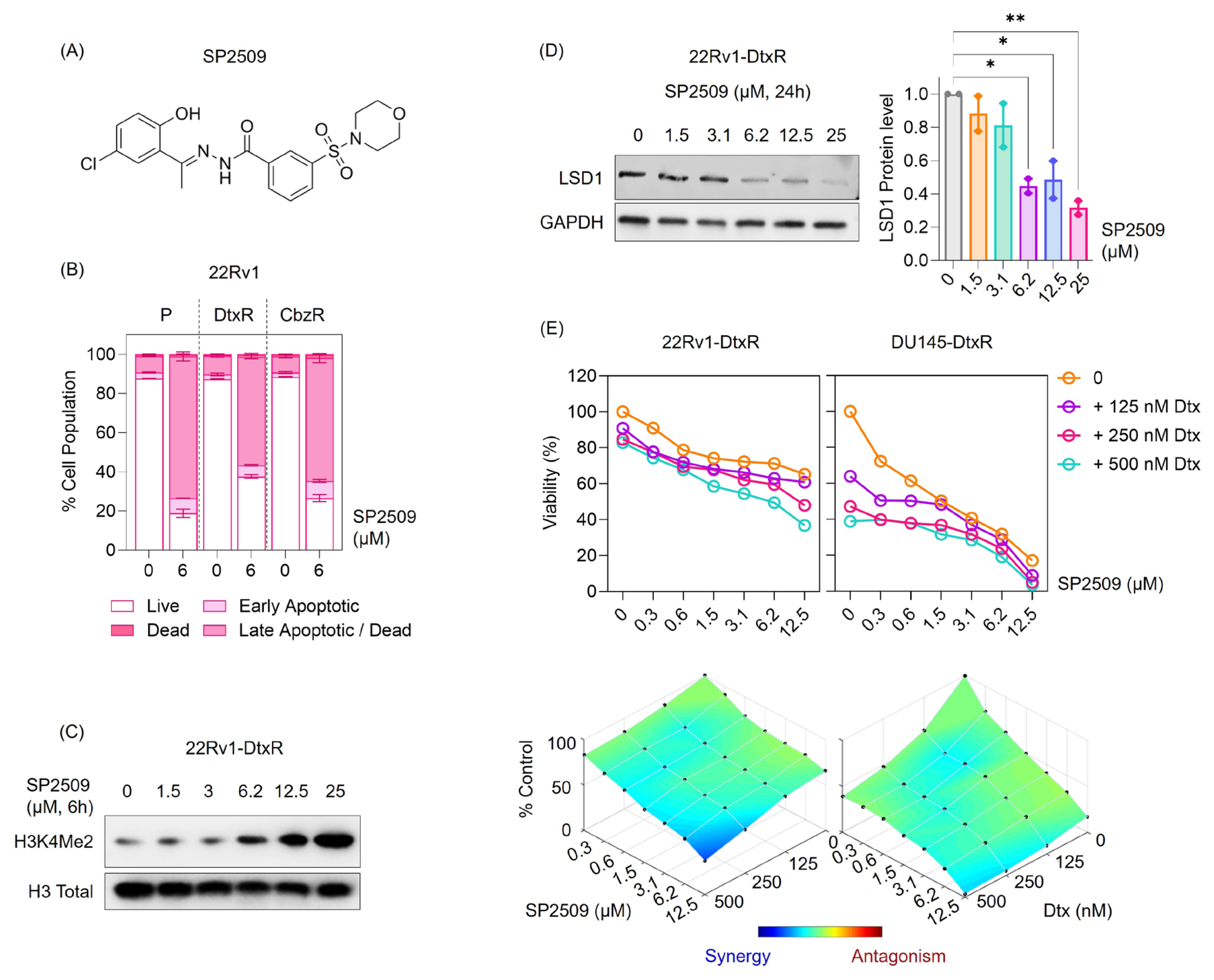

Supplement: Supplemental Figure — Cellular effects of SP2509. (A) Chemical structure of SP2509. (B) Annexin V/PI flow cytometry profiles showing redistribution from live to early/late apoptotic and dead fractions following SP2509 treatment (6 μM, 72 h) in 22Rv1 parental (P), DtxR, and CbzR cells. Data represent mean ± SEM from two replicates. (C) Western blot detection of H3K4Me2 following histone extraction from 22Rv1-DtxR cells treated with increasing concentrations of SP2509 (1.5–25 μM, 6 h). H3 Total was used as the loading control. (D) Left: Western blot detection of LSD1 protein in DU145-DtxR cells treated with SP2509 (1.5–25 μM, 24 h). GAPDH was the loading control. Rigt: Quantification of LSD1 protein levels normalized to GAPDH, shown relative to untreated control (0). Statistical analysis was performed using one-way ANOVA followed by Dunnett’s multiple comparisons test. Significance levels: p < 0.05 (*), p < 0.01 (**) compared with corresponding ‘0 μM’ controls. (E) Upper panel: Dose-response curves of 22Rv1-DtxR and DU145-DtxR cells treated with SP2509 alone (0, 0.3–12.5 μM) or in combination with fixed doses of Dtx (125–500 nM). Lower panel: Interaction surface generated with Combenefit (HSA model) for the “SP2509 × Dtx” combination. Heatmap colors indicate the interaction effect (blue, synergy; red, antagonism). [file tjb-49-07-757s1.tif]
